# Supplementary figures and images for: Similarity thresholds used in DNA sequence assembly from short reads can reduce the comparability of population histories across species
Source: PeerJ. 2015 Apr 21;3:e895. doi: 10.7717/peerj.895 (PMC4411482; doi:10.7717/peerj.895)

Total Bases in Alignments

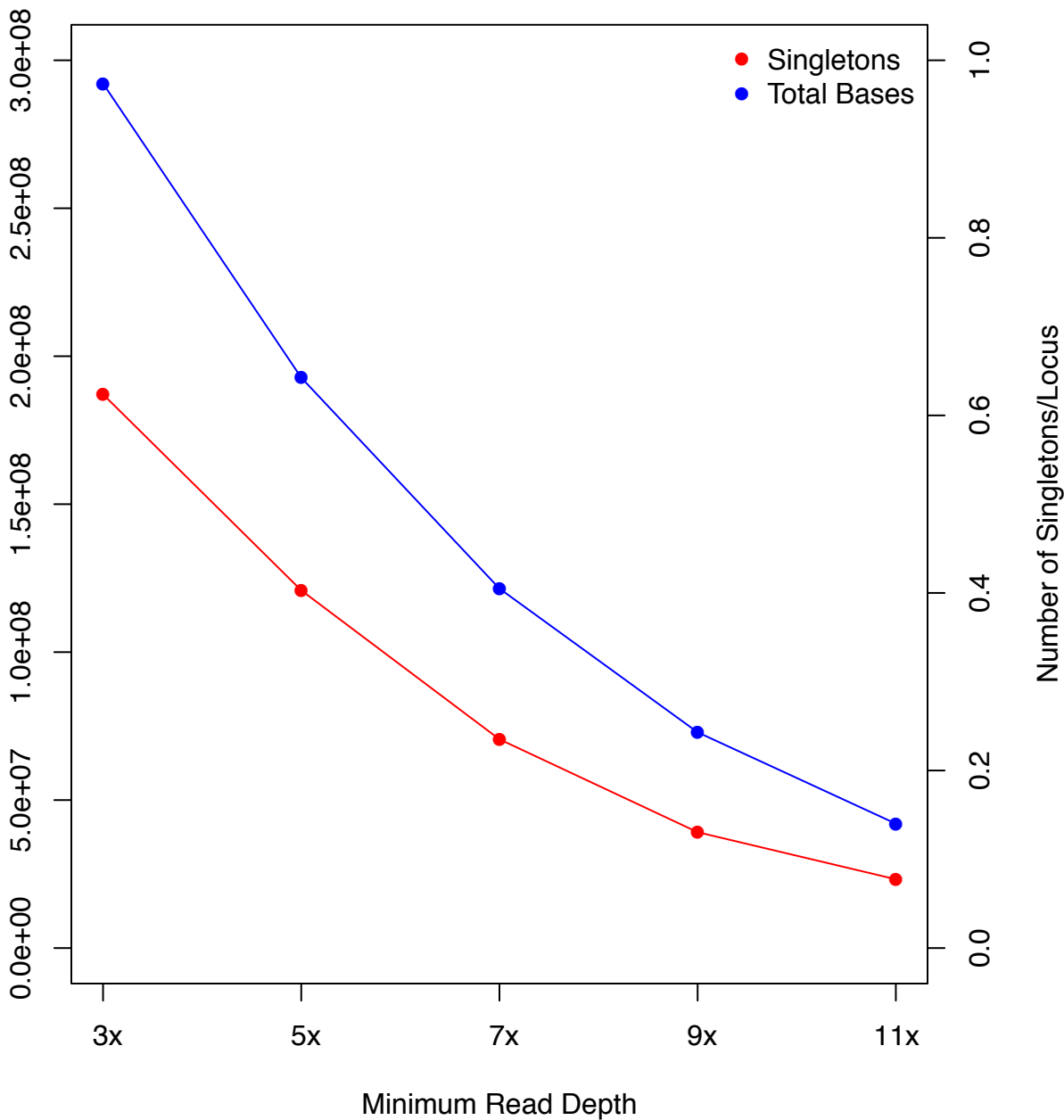

Supplement: Figure S1 — Adjusting minimum read depth used to form alleles in Stacks affects both the total number of bases in a dataset and the number of singleton alleles, the latter serving as a proxy for the frequency of errors. We selected a read depth of 7× for further analyses because, among the settings examined, it appeared to balance the inclusion of potential errors with the total size of the data matrix. [file peerj-03-895-s001.pdf]

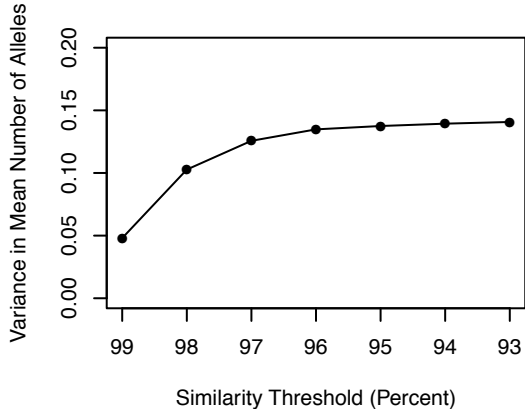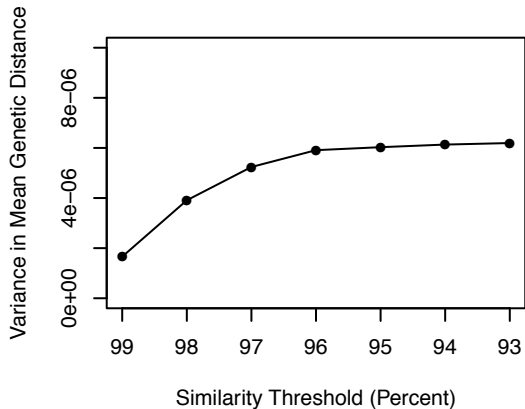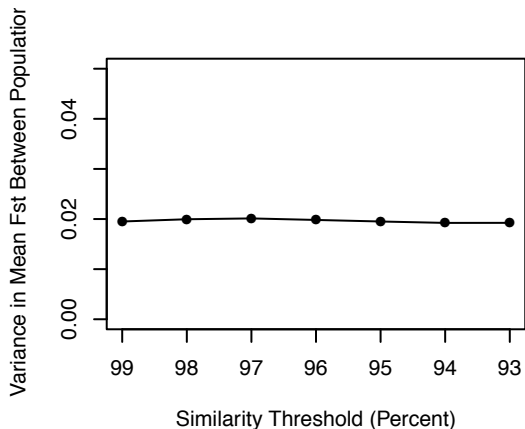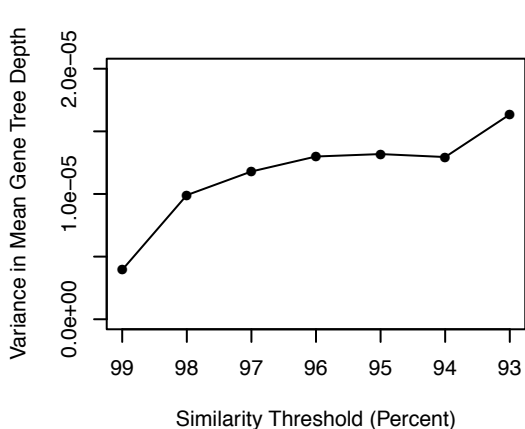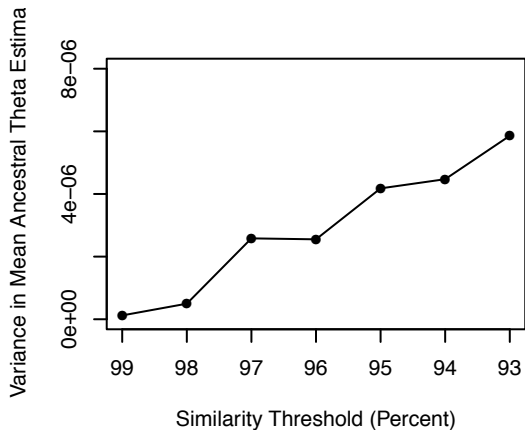

Supplement: Figure S2 — Variance across the four study species in mean number of alleles and mean estimates of phylogeographic and population genetic parameters (aside from Fst) varies depending on the similarity threshold applied. [file peerj-03-895-s002.pdf]

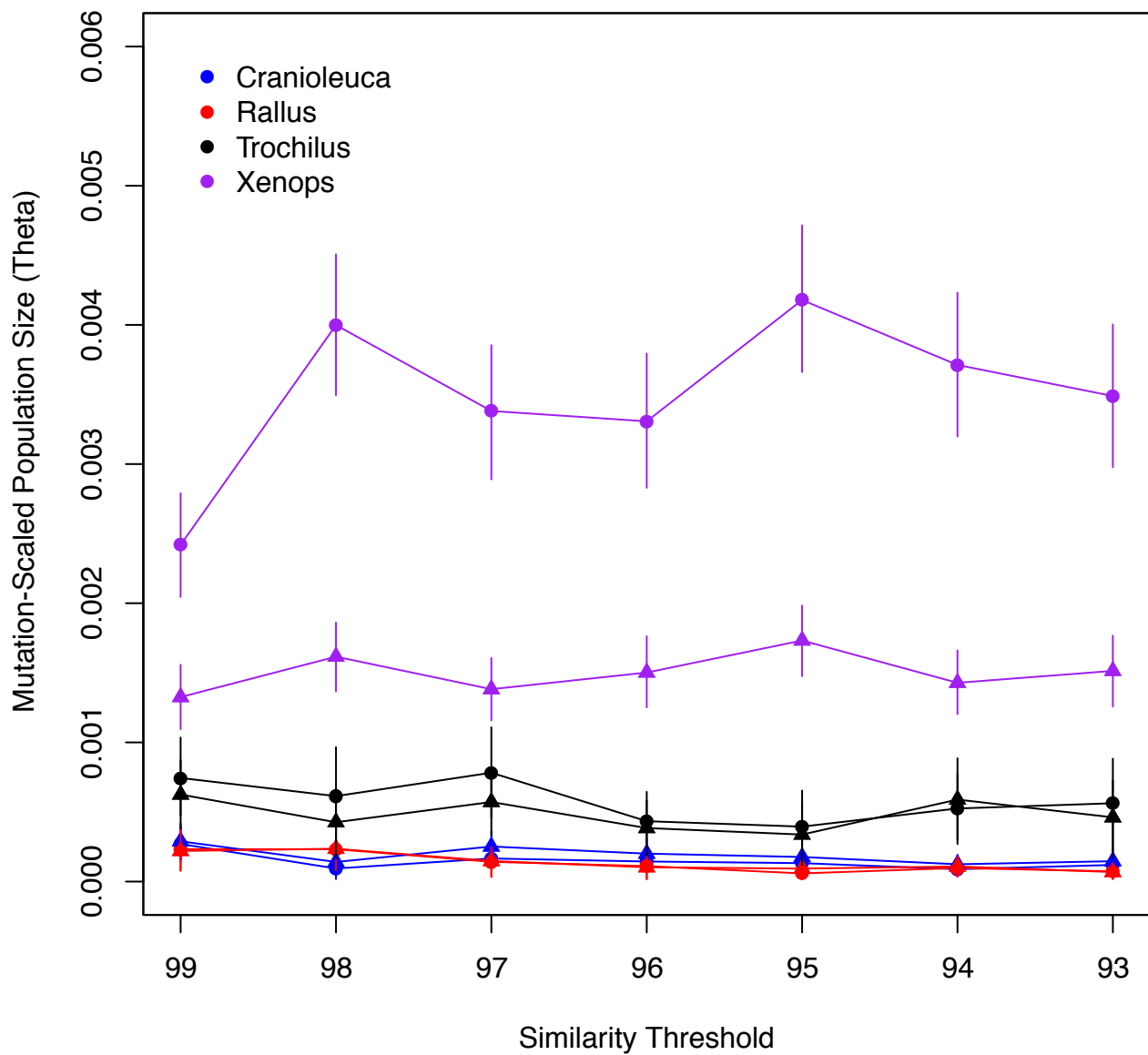

Supplement: Figure S3 — Mean values of mutation-scaled effective population size (theta) of the two extant populations did not show an association with similarity threshold for any species. Circles and triangles are used to distinguish the two daughter populations from each species. [file peerj-03-895-s003.pdf]

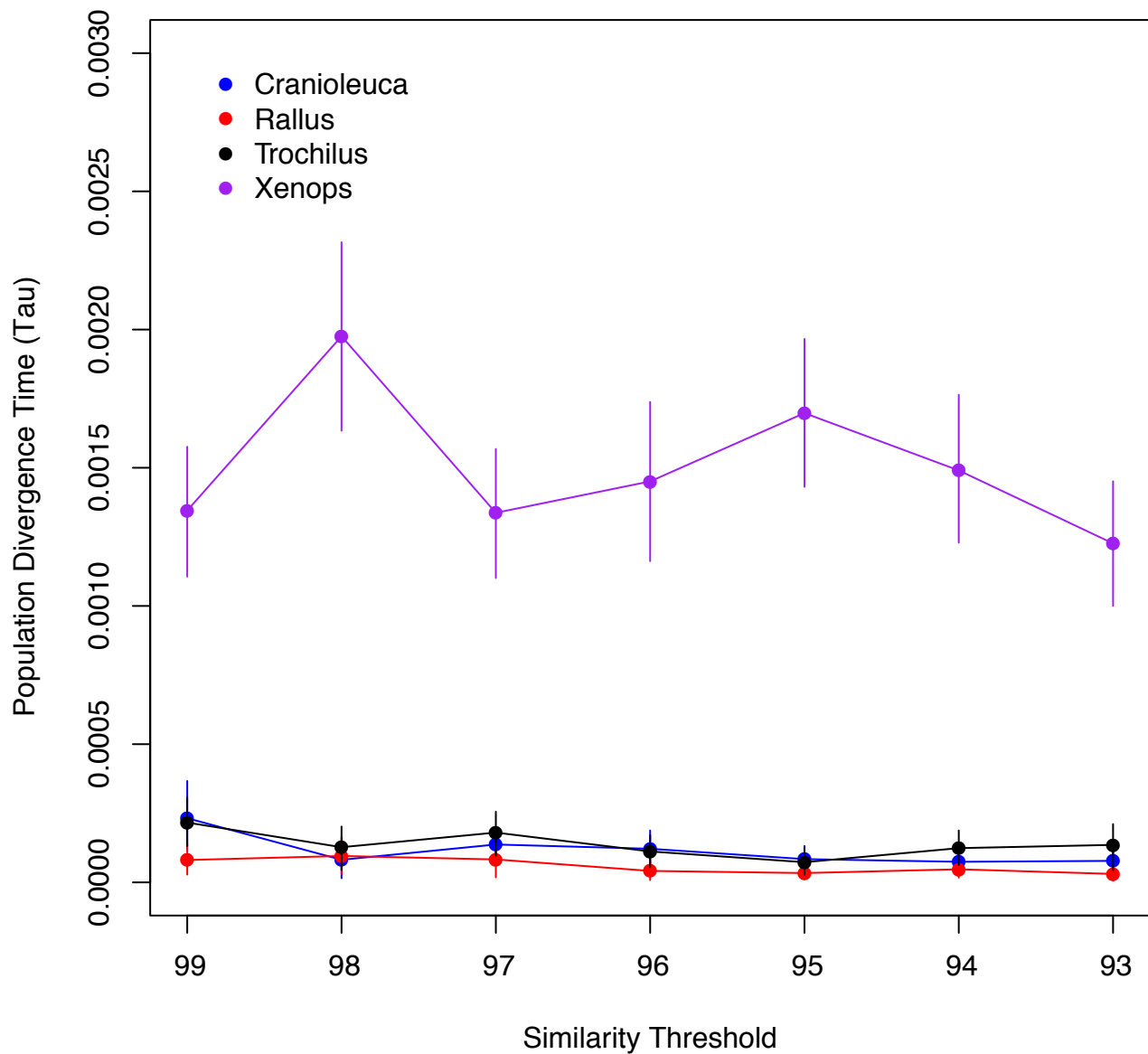

Supplement: Figure S4 — Mean values of population divergence time (tau) between the two extant populations did not show an association with similarity threshold for any species. [file peerj-03-895-s004.pdf]
