# Supplementary material for: Similarity thresholds used in DNA sequence assembly from short reads can reduce the comparability of population histories across species
Source: PeerJ. 2015 Apr 21;3:e895. doi: 10.7717/peerj.895 (PMC4411482; doi:10.7717/peerj.895)
Supplement: Table S1 [file peerj-03-895-s005.docx]

|  | **Population** | **Genus** | **Species** | **Subspecies** | **Museum** | **Tissue #** |
| --- | --- | --- | --- | --- | --- | --- |
| 1 | 1 | *Cranioleuca* | *antisiensis* | *antisiensis* | LSUMZ | 67126 |
| 2 | 1 | *Cranioleuca* | *antisiensis* | *antisiensis* | LSUMZ | 67136 |
| 3 | 1 | *Cranioleuca* | *antisiensis* | *antisiensis* | LSUMZ | 67177 |
| 4 | 1 | *Cranioleuca* | *antisiensis* | *antisiensis* | LSUMZ | 67243 |
| 5 | 2 | *Cranioleuca* | *baroni* | *capitalis* | LSUMZ | 72753 |
| 6 | 2 | *Cranioleuca* | *baroni* | *capitalis* | LSUMZ | 72754 |
| 7 | 2 | *Cranioleuca* | *baroni* | *capitalis* | LSUMZ | 72756 |
| 8 | 2 | *Cranioleuca* | *baroni* | *capitalis* | LSUMZ | 72759 |
| 9 | 1 | *Rallus* | *crepitans* | *saturatus* | LSUMZ | 63400 |
| 10 | 1 | *Rallus* | *crepitans* | *saturatus* | LSUMZ | 63404 |
| 11 | 1 | *Rallus* | *crepitans* | *saturatus* | LSUMZ | 63475 |
| 12 | 1 | *Rallus* | *crepitans* | *saturatus* | LSUMZ | 63477 |
| 13 | 2 | *Rallus* | *elegans* | *elegans* | LSUMZ | 63464 |
| 14 | 2 | *Rallus* | *elegans* | *elegans* | LSUMZ | 63467 |
| 15 | 2 | *Rallus* | *elegans* | *elegans* | LSUMZ | 63471 |
| 16 | 2 | *Rallus* | *elegans* | *elegans* | LSUMZ | 63473 |
| 17 | 1 | *Trochilus* | *polytmus* | *polytmus* | USNM | 4134 |
| 18 | 1 | *Trochilus* | *polytmus* | *polytmus* | USNM | 4136 |
| 19 | 1 | *Trochilus* | *polytmus* | *polytmus* | USNM | 4147 |
| 20 | 1 | *Trochilus* | *polytmus* | *polytmus* | USNM | 4160 |
| 21 | 2 | *Trochilus* | *polytmus* | *scitulus* | USNM | 3914 |
| 22 | 2 | *Trochilus* | *polytmus* | *scitulus* | USNM | 3915 |
| 23 | 2 | *Trochilus* | *polytmus* | *scitulus* | USNM | 3931 |
| 24 | 2 | *Trochilus* | *polytmus* | *scitulus* | USNM | 3935 |
| 25 | 1 | *Xenops* | *minutus* | *mexicanus* | KUMNH | 2044 |
| 26 | 1 | *Xenops* | *minutus* | *mexicanus* | LSUMZ | 60935 |
| 27 | 1 | *Xenops* | *minutus* | *littoralis* | LSUMZ | 2209 |
| 28 | 1 | *Xenops* | *minutus* | *littoralis* | LSUMZ | 11948 |
| 29 | 2 | *Xenops* | *minutus* | *obsoletus* | LSUMZ | 4244 |
| 30 | 2 | *Xenops* | *minutus* | *obsoletus* | LSUMZ | 6862 |
| 31 | 2 | *Xenops* | *minutus* | *obsoletus* | LSUMZ | 9026 |
| 32 | 2 | *Xenops* | *minutus* | *obsoletus* | FMNH | 433364 |
| *NOTE: Museums are Field Museum of Natural History (FMNH), University of Kansas Natural History Museum (KUNHM), Louisiana State University Museum of Natural Science (LSUMZ), and Smithsonian Institution National Museum of Natural History (USNM)* | | | | | | |
