# Supplementary material for: Similarity thresholds used in DNA sequence assembly from short reads can reduce the comparability of population histories across species
Source: PeerJ. 2015 Apr 21;3:e895. doi: 10.7717/peerj.895 (PMC4411482; doi:10.7717/peerj.895)
Supplement: Table S2 [file peerj-03-895-s006.docx]

| **Parameter** | **Setting** |
| --- | --- |
| Disable recording SQL data in the database (-S) |  |
| Number of threads (-T) | 8 |
| Minimum stack depth (-m) | 10 |
| Distance allowed between stacks (-M) | 1,2,3,4,5,6,7 |
| Distance allowed between catalog loci (-n) | 1,2,3,4,5,6,7 |
| Remove highly repetitive RAD-Tags (-t) |  |
| Maximum distance allowed to align secondary reads (-N) | 0 |
| Disable calling haplotypes from secondary reads (-H) |  |
| Maximum number of stacks at a single de novo locus (--max_locus_stacks) | 3 |
