# Supplementary material for: Similarity thresholds used in DNA sequence assembly from short reads can reduce the comparability of population histories across species
Source: PeerJ. 2015 Apr 21;3:e895. doi: 10.7717/peerj.895 (PMC4411482; doi:10.7717/peerj.895)
Supplement: Table S3 [file peerj-03-895-s007.docx]

|  | **chi-squared value** | **df** | **p-value** |
| --- | --- | --- | --- |
| *Cranioleuca* | 2682.455 | 6 | 2.20E-16 |
| *Rallus* | 1007.52 | 6 | 2.20E-16 |
| *Trochilus* | 2301.125 | 6 | 2.20E-16 |
| *Xenops* | 15267.58 | 6 | 2.20E-16 |
