# Supplementary material for: Similarity thresholds used in DNA sequence assembly from short reads can reduce the comparability of population histories across species
Source: PeerJ. 2015 Apr 21;3:e895. doi: 10.7717/peerj.895 (PMC4411482; doi:10.7717/peerj.895)
Supplement: Table S4 [file peerj-03-895-s008.docx]

|  | ***Cranioleuca*** | ***Rallus*** | ***Trochilus*** | ***Xenops*** |
| --- | --- | --- | --- | --- |
| 99 | 0.0022 | 0.0020 | 0.0011 | 0.0023 |
| 98 | 0.0028 | 0.0028 | 0.0016 | 0.0031 |
| 97 | 0.0030 | 0.0028 | 0.0016 | 0.0033 |
| 96 | 0.0029 | 0.0029 | 0.0015 | 0.0033 |
| 95 | 0.0031 | 0.0029 | 0.0016 | 0.0033 |
| 94 | 0.0031 | 0.0030 | 0.0017 | 0.0034 |
| 93 | 0.0031 | 0.0033 | 0.0018 | 0.0035 |
